# Cost of Manufacturing for Recombinant Snakebite Antivenoms

**DOI:** 10.3389/fbioe.2020.00703

**Published:** 2020-07-10

**Authors:** Timothy Patrick Jenkins, Andreas Hougaard Laustsen

**Affiliations:** Department of Biotechnology and Biomedicine, Technical University of Denmark, Lyngby, Denmark

**Keywords:** next-generation antivenoms, cost of manufacture, snakebite, envenoming, toxin neutralization, antivenom manufacture, human monoclonal antibodies, alternative protein scaffolds

## Abstract

Snakebite envenoming is a neglected tropical disease that affects millions of people across the globe. It has been suggested that recombinant antivenoms based on mixtures of human monoclonal antibodies, which target key toxins of medically important snake venom, could present a promising avenue toward the reduction of morbidity and mortality of envenomated patients. However, since snakebite envenoming is a disease of poverty, it is pivotal that next-generation therapies are affordable to those most in need; this warrants analysis of the cost dynamics of recombinant antivenom manufacture. Therefore, we present, for the first time, a bottom-up analysis of the cost dynamics surrounding the production of future recombinant antivenoms based on available industry data. We unravel the potential impact that venom volume, abundance of medically relevant toxins in a venom, and the molecular weight of these toxins may have on the final product cost. Furthermore, we assess the roles that antibody molar mass, manufacturing and purification strategies, formulation, antibody efficacy, and potential cross-reactivity play in the complex cost dynamics of recombinant antivenom manufacture. Notably, according to our calculations, it appears that such next-generation antivenoms based on cocktails of monoclonal immunoglobulin Gs (IgGs) could be manufacturable at a comparable or lower cost to current plasma-derived antivenoms, which are priced at USD 13-1120 per treatment. We found that monovalent recombinant antivenoms based on IgGs could be manufactured for USD 20-225 per treatment, while more complex polyvalent recombinant antivenoms based on IgGs could be manufactured for USD 48-1354 per treatment. Finally, we investigated the prospective cost of manufacturing for recombinant antivenoms based on alternative protein scaffolds, such as DARPins and nanobodies, and highlight the potential utility of such scaffolds in the context of low-cost manufacturing. In conclusion, the development of recombinant antivenoms not only holds a promise for improving therapeutic parameters, such as safety and efficacy, but could possibly also lead to a more competetive cost of manufacture of antivenom products for patients worldwide.

## Introduction

The World Health Organization recently reclassified snakebite envenoming as a Category A Neglected Tropical Disease and developed a strategy for reducing the morbidity and mortality for snakebite victims worldwide (Chippaux, 2017; Williams et al., 2019). As an important part of this strategy, research and development on improved snakebite envenoming therapies is recommended. In this relation, a promising avenue that has gained interest in recent years, is the use of recombinant antivenoms based on carefully designed mixtures of human monoclonal antibodies targeting key toxins of medically important snake venoms (Laustsen, 2016). Some of the hypothesized benefits of using recombinant antivenoms include a reduced propensity to cause adverse reactions in patients and a higher content of therapeutically active antibodies (Kini et al., 2018). Additionally, recombinant antivenoms have also been hypothesized to be manufacturable at low cost (Laustsen et al., 2016, 2017), which is an important parameter for therapies against neglected tropical diseases. However, one of the challenges that sets snakebite envenoming aside from other indications that are treatable with antibodies is that exceptionally high amounts of antibodies are needed for effective treatment (Laustsen, 2019). Therefore, the cost of manufacture should be a key focal point for recombinant antivenom developers (Laustsen and Dorrestijn, 2018; Knudsen et al., 2019). Yet, so far, this has remained largely unexplored. Currently, the only estimates are based on top-down calculations built on limited knowledge derived from conventional polyclonal plasma-derived antivenoms paired with data from general industrial manufacture of monoclonal antibodies (Laustsen et al., 2017). However, with recent developments in the field of recombinant antivenom research and reports of monoclonal antibodies being effective at low dose in neutralizing key toxins in different animal venoms (Richard et al., 2013; Knudsen and Laustsen, 2018; Laustsen et al., 2018), it is now possible to perform a more fine-grained estimation of the prospective cost of manufacture for recombinant antivenoms. Hence, here we present such estimates for the cost of manufacture for future recombinant antivenoms based on bottom-up calculations, which have the benefit over top-down calculations that more real-life data on antibody efficacy and venom yields can be incorporated. We present different antivenom cost scenarios for vipers and elapids that either inject large or small amounts of venoms, as well as we estimate the manufacturing costs for polyvalent recombinant antivenoms covering multiple snake species. Finally, we compare the theoretical cost of manufacture for the active pharmaceutical ingredient (API) with the cost of manufacture for the final drug product (FDP), as well as explore the relation between cost of manufacture and the molecular sizes of different antibody formats with the purpose of highlighting the influence of the number of toxin binding sites per mass unit for different types of antibodies.

## Materials and Methods

### Venoms Included in This Study and Definition of Key Toxins

In this study, we analyzed the theoretical cost of manufacture for recombinant antivenom production for 17 different snake venoms from Asia, Africa, Australia, North America, Central America, and South America. For all of these species, we collated data on the maximum venom yields (dry weight) recorded for each species (*m*_yield_), protein composition (based on proteomics studies), and estimated percentage of toxins in a given venom that needs to be neutralized for successful clinical outcome (*r*_% neutrali z ed_; Table 1). *r*_% neutralized_ was based on the combined percentage of snake venom metalloproteinases (SVMPs, including sub-families SVMP PI and PIII), snake venom serine proteinases (SVSPs), phospholipases A_2_ (PLA_2_s), three-finger toxins (3FTxs), β-bungarotoxins, dendrotoxins, C-type lectins, and disintegrins present in the venom; *r*_% neutralized_ was set conservatively at the highest value realistically possible.

**TABLE 1:** Venoms Included in Our Costing Analyses for Recombinant Antivenoms.

Species	*m*_yield_	Venom composition (medically relevant toxins)	*r*_% neutralized_	References
*Naja naja* (proteomics: East India; yield: India)	0.169 g	PLA_2_s (11.4%) and 3FTxs (63.8%)	75.2%	Broad et al., 1979; Dutta et al., 2017
*Echis carinatus* (proteomics: India; yield: Iran)	0.04 g	SVMPs (45.4%), SVSPs (0.3%), disintegrins (14%), C-type lectins (23.9%), and PLA_2_s (10.9%)	94.5%	Latifi, 1984; Patra et al., 2017
*Bungarus caeruleus* (proteomics: South India; yield:Sri Lanka)	0.024 g	SVMPs (4.8%), SVSPs (0.1%), PLA_2_s (24.7%), 3FTxs (48.3%), and β-bungarotoxins (12.9%)	90.8%	Williams et al., 2011; Patra et al., 2019
*Daboia russelii* (proteomics: East India; yield: Sri Lanka)	0.115 g	SVMPs (18.8%), SVSPs (14.1%), disintegrins (1.8%), C-type lectins (11.6%), and PLA_2_s (21.9%)	68%	Williams et al., 2011; Kalita et al., 2018
*Pseudechis australis* (proteomics: Australia; yield: Australia)	0.787 g	SVMPs (53%) and PLA_2_s (18.5%)	71.5%	Mirtschin et al., 2006; Georgieva et al., 2011
*Micrurus nigrocinctus* (proteomics: Costa Rica; yield: Costa Rica)	0.008 g	SVMPs (4.3%), PLA_2_s (48%), C-type lectins (2.2%), and 3FTxs (38%)	92.5%	Chacón et al., 2012; Fernández et al., 2011
*Crotalus adamanteus* (proteomics: United States; yield: Florida)	0.41 g	SVMPs (16%), SVSPs (25%), C-type lectins (5%), and PLA_2_s (28%)	97%	Broad et al., 1979; Margres et al., 2014
*Bothrops atrox* (proteomics: South America; yield: South America - average)	0.2 g	SVMPs (83%; PI 14% and PII 69%), SVSPs (4.5%), disintegrins (0.2%), C-type lectins (0.1%), and PLA_2_s (10%)	97.8%	O’Shea, 2005; Calvete et al., 2011
*Bitis arietans* (proteomics: Africa; yield: Africa)	0.29 g	SVMPs (38.5%), SVSPs (19.5%), disintegrins (17.8%), C-type lectins (13.2%), and PLA_2_s (4.3%)	93.2%	Juárez et al., 2006; Mirtschin et al., 2006
*Bitis gabonica* (proteomics: Africa; yield: Africa)	0.24 g	SVMPs (22.9%), SVSPs (26.4%), C-type lectins (14.3%), and PLA_2_s (11.4%)	75%	Marsh and Whaler, 1984; Calvete et al., 2007a
*Echis ocellatus* (proteomics: Nigeria; yield: Nigeria)	0.016 g	SVMPs (61.4%; PI 6.3% and PIII 55.1%), SVSPs (1.9%), disintegrins (6.8%), C-type lectins (7%), and PLA_2_s (11.7%)	89.8%	Wagstaff et al., 2009; Williams et al., 2011
*Echis leucogaster* (*E. ocellatus used as proxy;* proteomics: Nigeria; yield: Nigeria)	0.016 g	SVMPs (61.4%; PI 6.3% and PIII 55.1%), SVSPs (1.9%), disintegrins (6.8%), C-type lectins (7%), and PLA_2_s (11.7%)	89.8%	Wagstaff et al., 2009; Williams et al., 2011
*Dendroaspis polylepis* (proteomics: Tanzania; yield: East Africa)	0.026 g	SVMPs (3.2%), 3FTxs (31%), and dendrotoxins (20%)	54.2%	Williams et al., 2011; Petras et al., 2016
*Dendroaspis jamesoni* (proteomics: Cameroon; yield: Africa)	0.12 g	3FTxs (80%), and dendrotoxins (12.5%)	92.5%	O’Shea, 2005; Ainsworth et al., 2018
*Dendroaspis viridis* (proteomics: Togo; yield: Africa)	0.1 g	3FTxs (78%), and dendrotoxins (2.1%)	80.1%	O’Shea, 2005; Ainsworth et al., 2018
*Naja haje* (proteomics: Morocco; yield: Africa)	0.3 g	SVMPs (9%), PLA_2_s (4%), and 3FTxs (60%)	73%	O’Shea, 2005; Malih et al., 2014
*Naja nigricollis* (proteomics: W Africa; yield: Nigeria)	0.362 g	SVMPs (2.4%; PIII 2.4%), PLA_2_s (21.9%), and 3FTxs (73.3%)	97.6%	Williams et al., 2011; Petras et al., 2011
*Naja melanoleuca* (proteomics: Uganda; yield: Africa)	1.1 g	SVMPs (9.7%), PLA_2_s (12.9%), and 3FTxs (57.1%)	79.7%	Mirtschin et al., 2006; Lauridsen et al., 2017

*The table includes species names and origin of specimens used to determine the proteome and yield, as well as maximum venom yields (dry weight) recorded for each species (*m*_*yield*_), protein composition of each venom, and estimated percentage of toxins that need to be neutralized for successful clinical outcome (*r*_% *neutralized*_). The toxin families considered medically relevant in relation to these venoms include snake venom metalloproteinases (SVMPs, including sub-families SVMP PI and PIII), snake venom serine proteinases (SVSPs), phospholipases A_2_ (PLA_2_s), three-finger toxins (3FTxs), β-bungarotoxins, dendrotoxins, C-type lectins, and disintegrins.*

### Average Molar Mass of Toxins

To calculate the average molar mass (M) of the medically relevant toxins in each venom, the average molar mass of each toxin family (M_Tox_; Table 2) was based on an average value; specific molecular weight data for each toxin in a proteome is often not available, hence the values were fixed according to parameters accepted by the scientific community (Strong et al., 1976; Harvey, 2001; Calvete et al., 2007b; Serrano, 2012; Wong et al., 2016; Ferraz et al., 2019). M was then multiplied with its relative abundance (Ra) in each respective venom. Upon summation of all values and division by the total Ra of all toxin families combined (Ra_Sum_), an average molar mass for each venom was generated (Eq. 1).

**TABLE 2:** Average Molar Masses of All Toxin Families Considered to be Medically Relevant.

TOXIN family	Molar mass	References
SVMP	50 kDa	Ferraz et al., 2019
SVMP (PI)	25 kDa	Ferraz et al., 2019
SVMP (PIII)	80 kDa	Ferraz et al., 2019
SVSP	30 kDa	Serrano, 2012
PLA_2_	14 kDa	Ferraz et al., 2019
3FTx	8 kDa	Wong et al., 2016
β-bungarotoxin	16 kDa	Strong et al., 1976
Dendrotoxins	7 kDa	Harvey, 2001
C-type lectins	28 kDa	Calvete et al., 2007a
Disintegrins	9.5 kDa	Calvete et al., 2007a

*This includes snake venom metalloproteinases (SVMPs, including sub-families SVMP PI and PIII), snake venom serine proteinases (SVSPs), phospholipases A_2_ (PLA_2_s), three-finger toxins (3FTxs), β-bungarotoxins, dendrotoxins, C-type lectins, and disintegrins.*

(1) MV⁢e⁢n⁢o⁢m=MT⁢o⁢x⁢ 1×RaT⁢o⁢x⁢ 1+MT⁢o⁢x⁢ 2×R⁢aT⁢o⁢x⁢ 2+⋯⁢MT⁢o⁢x⁢x×R⁢aT⁢o⁢x⁢xRaSum

### Calculating the Amount of Antibodies Required for Neutralization for a Monovalent Antivenom

With the previously mentioned variables, we proceeded to calculate the approximate amount of antibodies (Abs) required (m_Ab required_; grams) to neutralize each of the venoms included in this study (Eq. 2). M_Ab_ designates the molar mass of antibody [for immunoglobulin G (IgG) this is 150 kDa]. However, we also wanted to account for fluctuations in venom injected in any given bite (*r*_% *injected*_). Therefore, we included three possible bite scenarios, i.e., 25% of *m*_yield_, 50% of *m*_yield_, and 100% of *m*_yield_. We also included three different estimates of how many antibodies would be required to neutralize each toxin, expressed as ratios between toxin-to-antibody (*R*_*Tox:Ab*_), i.e., 2:1, 1:1, and 1:3. These ratios were chosen based on previously reported data demonstrating that effective antibodies can neutralize medically relevant toxins at these ratios (Richard et al., 2013; Laustsen et al., 2018; Silva et al., 2018; Calderon et al., 2020).

(2) $$m_{A\,b\,r\,e\,q\,u\,i\,r\,e\,d}=\frac{m_{y\,i\,e\,l\,d}\times r_{\%neutralised}\times r_{\%injected}}{M_{V\,e\,n\,o\,m}}\;\times M_{A\,b}\times R_{T\,o\,x:A\,b}$$

### Antibody Manufacturing Strategies

**FIGURE 1:**
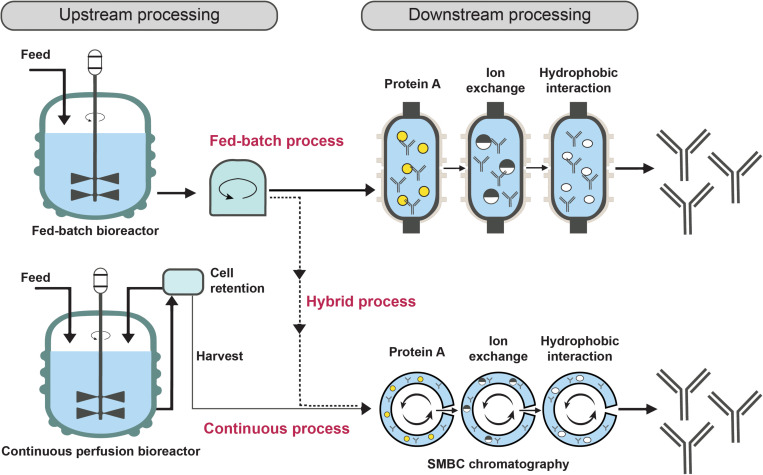
Three different antibody manufacturing process strategies. The fed-batch process involves the one-off supply of nutrients for the CHO cells for a complete cultivation process. Subsequently, the antibodies are harvested and purified via single-batch chromatography. This is not the case for the continuous perfusion process, where cells are retained while the growth medium containing the antibodies is continuously replaced with fresh medium in a perfusion bioreactor. Subsequently, the media undergoes simulated moving bed chromatography (SMBC), where the chromatographic processes are performed via a continuous process as well. The hybrid process is a combinantion of the two previous approaches in that it involves the use of a fed-batch bioreactor followed by SMBC instead of single-batch chromatography.

Different manufacturing approaches can have a significant impact on the manufacturing costs for recombinant monoclonal antibodies (Cost_Ab_). In this study, estimated costs for three types of manufacturing processes based on Chinese Hamster Ovary (CHO) cell cultivation were employed: The fed-batch process (nutrients for the CHO cells are supplied for a complete manufacturing process, followed by harvest of the entire batch), the hybrid process (cultivation is performed in a fed-batch bioreactor, followed by continuous or semi-continuous purification of the produced antibodies), and the continuous perfusion process (cultivated cells are retained in the bioreactor, while the growth medium containing the antibodies is continuously substituted with fresh medium in a perfusion bioreactor; the used medium undergoes a continuous or semi-continuous purification process in order to isolate the antibodies; Figure 1; Hammerschmidt et al., 2014; Walsh, 2014). Each manufacturing method was then combined with a downstream process based on either chromatographic or caprylic acid purification. The approximate costs for each process can be found in Table 3 derived from Laustsen et al. (2017), which assume an annual production volume of 500 kg of antibodies. The cost estimates for the different manufacturing strategies are based on available industrial data as well as prior discussions with five independent experts from five different companies in Germany, Denmark, and the United Kingdom (Farid, 2007; Rasmussen et al., 2012; Hammerschmidt et al., 2014; Walsh, 2014; Klutz et al., 2015; Laustsen et al., 2017). The assumed volume was based on a previous assessment for what the need for a sub-Saharan antivenom would be to establish the approximate scale of manufacture. Here, it was chosen to also use the manufacturing data at this scale to allow for a direct comparison with the previously reported top-down cost assessment for recombinant antivenoms (Laustsen et al., 2017). Depending on the manufacturing and purification process employed, the cost of the recombinant monoclonal antibodies will be either higher or lower. Therefore we calculated the exact cost impact each strategy would have on a recombinant antivenom. However, for final cost analyses of recombinant antivenoms, only the hybrid process combined with caprylic acid precipitation (hybrid_cap._) was employed, as it was projected to be the most cost-competitive approach and, thus, potentially most promising for future recombinant antivenom manufacture. It is noteworthy that whilst purification via caprylic acid is less expensive than chromatography, the latter can be employed to obtain a product of even higher purity.

**TABLE 3:** Cost estimates for different antibody manufacturing strategies, followed by either chromatographic or caprylic acid purification.

Cost_Ab_		Downstream process	
		
		Chromatography	Caprylic acid
	Fed-Batch	62 USD/g	46 USD/g
Upstream process	Hybrid	47 USD/g	33 USD/g
	Continuous perfusion	89 USD/g	42 USD/g

*These estimates assume an annual production volume of 500 kg of antibodies.*

### The Cost of Goods Manufactured of Active Pharmaceutical Ingredient (COGM_API_) and the Final Drug Product (COGM_FDP_)

When we calculate the product of Cost_Ab_ and m_*Ab required*_, we obtain the Cost of Goods Manufactured of Active Pharmaceutical Ingredient for a full treatment of a given snakebite (COGM_API_; Eq. 3).

(3) $$C\,O\,G\,M_{A\,P\,I}=C\,o\,s\,t_{A\,b}\times m_{A\,b\,r\,e\,q\,u\,i\,r\,e\,d}$$

We can then move on to calculate the Cost of Goods Manufactured for the Final Drug Product for a full treatment of a given snakebite (COGM_FDP_). For this, we used cost estimations for formulation and packaging, also known as Fill Finish, from a previous study (Laustsen et al., 2017). The authors estimated that for future recombinant antivenoms an average of four vials per treatment would be optimal for a treating clinician, since it allows for flexible dosing, and that the associated cost is five USD/vial, i.e., a total of USD 20, to the COGM_API_ (Eq. 4).

(4) C⁢O⁢G⁢MF⁢D⁢P=C⁢O⁢G⁢MA⁢P⁢I+U⁢S⁢D⁢ 20

### Cost of Recombinant Polyvalent Antivenoms

To estimate the cost of recombinant polyvalent antivenoms, we used two examples, namely a “simple” recombinant antivenom against the “Big 4” of India, i.e., *Naja naja*, *Echis carinatus*, *Daboia russelii*, and *Bungarus caerulus*, and a more complex one, including 10 different species. The latter is an estimation of the cost of manufacture for a recombinant (biosimilar) antivenom mimicking Sanofi Pasteur’s FAV-Afrique, a former high-quality polyvalent antivenom indicated for a wide range of species from sub-Saharan Africa. Here, our calculations include venoms from *Bitis arietans*, *B. gabonica*, *E. leucogaster*, *E. ocellatus*, *Dendroaspis polylepis*, *D. jamesoni*, *D. viridis*, *N. haje*, *N. nigricollis*, and *N. melanoleuca*. We assumed that *m*_yield_ equated to 50% of the maximum yield and also wanted to account for potential cross-reactivity of antibodies present in the hypothetical polyvalent antivenom (*r*_cr oss–react_) and, therefore, calculated COGM_API_ for three different scenarios with 0% cross-reactivity, 25% cross-reactivity, and 50% cross-reactivity for the antibodies included in the polyvalent recombinant antivenom. The estimations made toward cross-reactivity were based on the extremes of having either no cross-reactivity or a maximum of 50% cross-reactivity to capture the full spectrum of likely cross-reactivities. In the case, where no cross-reactivity is present, antibodies are needed for all toxins from all venoms. In the opposite case with 100% cross-reactivity, the antibodies needed for neutralizing the venom with the highest amount of toxins (*n*_venom max_) would (due to their cross-reactivity) be able to neutralize all other toxins from other venoms. Consequently, we can calculate the total antibodies (in mol) needed for neutralizing all venoms (*n*_Tox_). This is described by the following equation (Eq. 5):

$$n_{Tox}\;=n_{venom\;\;max\;}+\;\sum_{n_{venom\;min}}^{n_{venom\;max-1}}n_{venom\;\;x}\;\times \;(1-r_{cross-react})$$

Finally, COGM_FDP_ was calculated as described above. Here, *M*_Ab required_ was calculated using *n*_Tox_ and *M*_Ab_ (Eq 6).

$${\text{COGM}}_{\text{FDP,polyvalent}}=n_{Tox}\times M_{Ab}\times R_{Tox:Ab}\times Cost_{Ab,\;hybrid/cap}+USD\;20$$

### Costing Recombinant Antivenom Products Based on Alternative Antibody Formats

We also wanted to understand the impact of the small molar mass of alternative antibody formats, such as Fragment antigen binding (Fab; 50 kDa) and single-chain variable fragments (scFvs, 25 kDa), as well as alternative protein scaffolds, e.g., designed ankyrin repeat proteins (DARPins; 15 kDa), single-domain antibodies (nanobodies; 15 kDa), and Avimers (4 kDa). Although, some of these antibodies and binding proteins would likely be produced by different manufacturing processes, such as microbial fermentation, which may be even more cost-competitive, we decided to calculate the costs using the same parameters (with exception of M_Ab_) as for IgGs (33 USD/g) to facilitate direct cost comparison as a function of molecular sizes alone. To understand the impact that different molar masses can have on the COGM_FDP_ of a potentially expensive antivenom, we investigated this in the context of a recombinant FAV-Afrique biosimilar antivenom. Here, we assumed 1:1/1:3 toxin-to-antibody ratios and 25%/0% cross-reactivity to simulate an “expected” and a “worst-case” scenario, respectively.

## Results and Discussion

Understanding the dynamics of the manufacturing costs for next-generation antivenoms is pivotal toward developing effective, but also cost-competitive therapies for snakebite victims. Therefore, in the following, we present key variables to consider when assessing potential manufacturing costs for recombinant antivenoms using a bottom-up approach and conclude that they indeed represent a promising solution for next-generation snakebite envenoming therapy.

### Impact of Antibody Manufacturing Strategies and Formulation on the COGM for Recombinant Antivenom Therapy

**FIGURE 2:**
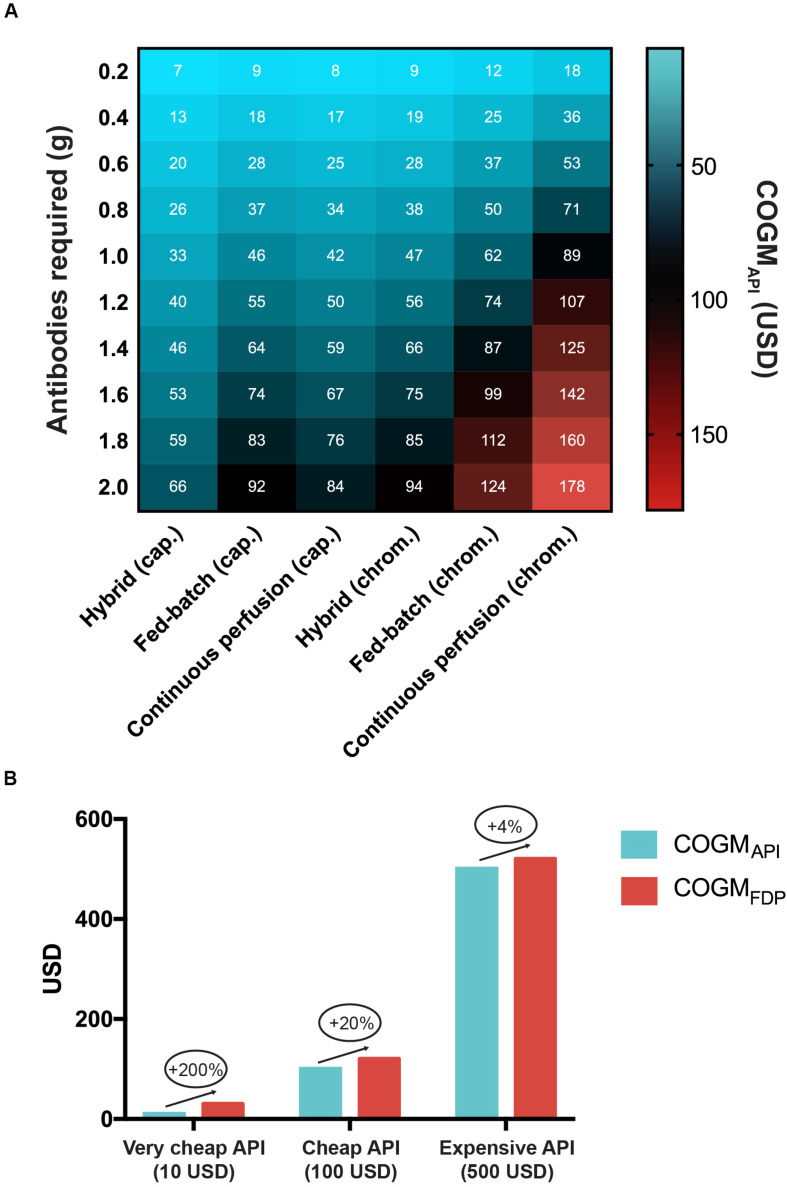
Cost of manufacture for recombinant antivenoms in relation to manufacturing process and treatment dose. **(A)** Cost impact of different manufacturing strategies in relation to how many grams of antibodies are required for a full antivenom treatment of a snakebite envenoming case. The three upstream processes included are the fed-batch process, the hybrid process, and the continuous perfusion process. Each upstream process was combined with either chromatographic or caprylic acid purification steps to calculate the respective Cost of Goods Manufactured of the Active Pharmaceutical Ingredient (COGM_API_) per treatment. The white numbers in the cells correspond to the exact COGM_API_ corresponding to that particular cell. **(B)** The impact of formulation on the final drug product (FDP) cost for very cheap, cheap, and expensive COGM_API_.

Many different strategies exist for the manufacture of recombinant antibodies. These utilize different downstream processes (such as chromatography and caprylic acid precipitation) and have different cost structures (Figure 2A). Based on available data from the scientific literature, and assuming an annual production volume of 500 kg of antibodies, the most costly manufacturing strategy for recombinant antibodies is continuous perfusion followed by chromatography, which is estimated to have a COGM_API_ of USD 89 per gram of antibody. Conversely, the most inexpensive strategy may involve a combination of the hybrid upstream process and caprylic acid purification (USD 33 per gram of antibody). This suggests that, from a cost perspective, the latter approach might be the most applicable for manufacture of recombinant antivenoms, for which cost is a major concern, as snakebite envenoming is most prevalent in rural impoverished areas of the tropics (Harrison et al., 2009). Our calculations also demonstrate the impact of formulation on the COGM_FDP_ (Figure 2B). Unsurprisingly, formulation costs barely affect the COGM_FDP_ of a product with a high COGM_API_, whilst it could lead to a 200% cost increase for antivenom products with a very low (USD 10) COGM_API_. Therefore, formulation costs are critical to take into consideration when manufacturing costs are low.

### How Molar Mass and Venom Quantity Affect COGM_API_

**FIGURE 3:**
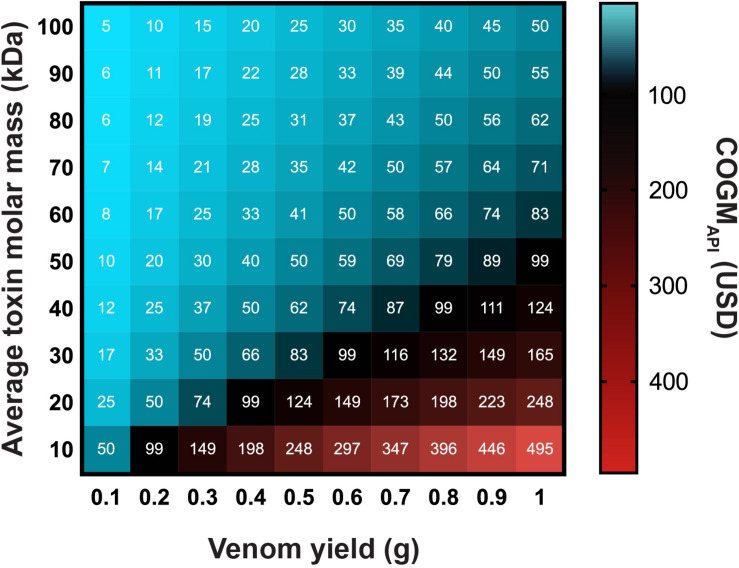
How the molecular weight and amount of venom to be neutralized affect the Cost of Goods Manufactured of the Active Pharmaceutical Ingredient (COGM_API_) for recombinant antivenoms. The heat map includes three variables, namely the amount of venom to be neutralized in grams, the average molecular mass of the venom toxins in kDa, and the COGM_API_ in USD. The white numbers in the cells correspond to the exact COGM_API_ corresponding to that particular cell.

The molar mass and amount of a given venom to be neutralized for a given snakebite case are also important cost-affecting factors (Figure 3). An amount of venom comprising toxins with lower molar masses will require more mols of antibodies for neutralization compared to the same amount of venom comprising toxins with higher molar masses. This is further amplified by the absolute amounts of venom being injected by a given snake. Consequently, bites from snakes that produce large volumes of venom comprising toxins with low average molar mass require the most antibodies and are, therefore, the most costly to neutralize. In contrast, bites from snakes that produce small volumes of venom comprising toxins with high average molar mass require the least antibodies and are the least costly to neutralize.

### COGM_FDP_ for Monovalent Recombinant Antivenoms

**FIGURE 4:**
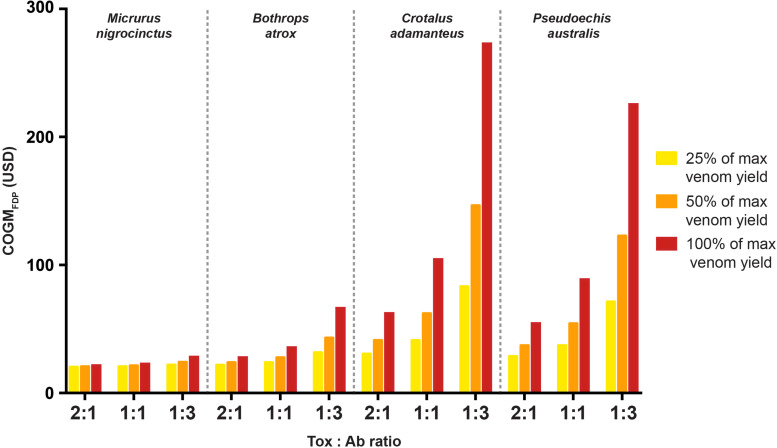
Cost of monovalent recombinant antivenoms against four representative species of venomous snakes. The calculations were conducted for three different toxin-to-antibody ratios (i.e., 2:1, 1:1, and 1:3) and assumed that either 25% (yellow), 50% (orange), or 100% (red) of the maximum venom yield needs to be neutralized. The calculations are for Cost of Goods Manufactured for the Final Drug Product for a full treatment of a given snakebite (COGM_FDP_) and, thus, include formulation and packaging costs.

Based on our previous calculations, we quantified the cost of four different putative monovalent recombinant antivenoms (Figure 4). These calculations were based on the assumption that the recombinant antibodies are manufactured via the hybrid process followed by caprylic acid precipitation. The calculations were conducted for three different toxin-to-antibody ratios (i.e., 2:1, 1:1, and 1:3) and assumed that either 25%, 50%, or 100% of the maximum venom yield (dry weight) is injected into a victim. Furthermore, to understand the above-mentioned cost dynamics of average venom toxin molar mass and venom amount, we included four snakes with different types of venoms and venom yields. The first snake (*M. nigrocinctus*) has a venom comprising toxins with a comparatively small average molar mass (13 kDa) and can only produce a very small volume of venom (0.008 g), the second snake (*B. atrox*) presents a venom comprising toxins with a large average molar mass (63 kDa), but still at a relatively small volume (0.2 g), the third snake (*C. adamanteus*) has a venom with a comparatively lower molar mass (23 kDa), but can produce 0.41 g of its venom, and finally *P. australis* venom has an average molar mass for its venom toxins of 40 kDa and can produce up to 0.79 g of the venom. It is notable that for both *M. nigrocinctus* and *B. atrox*, antibody efficacy and percentage of maximum venom yield injected had no major impact on the COGM_FDP_ of the respective monovalent antivenom (Figure 4), as the cost of formulation and packaging is the main cost driver. This was not the case when calculating the costs for the two other monovalent antivenoms against *C. adamanteus* and *P. australis*. Whilst the percentage of volume injected had a significant impact on the COGM_FDP_ for both antivenoms, the efficacy of the antibodies (reflected by the toxin-to-antibody ratio) had the largest effect on the cost. For instance, a monovalent recombinant antivenom of *C. adamanteus* that contained highly efficacious antibodies (i.e., one antibody per two toxins) would cost USD 29 per treatment when assuming 25% of max venom yield is injected and USD 54 when calculating with 100% of max venom yield injected. However, when assuming that three antibodies are required per toxin, the cost increases to USD 71 (25% of max venom yield injected) and USD 225 (100% of max venom yield injected), respectively.

### COGM_FDP_ for Two Polyvalent Recombinant Antivenoms

**FIGURE 5:**
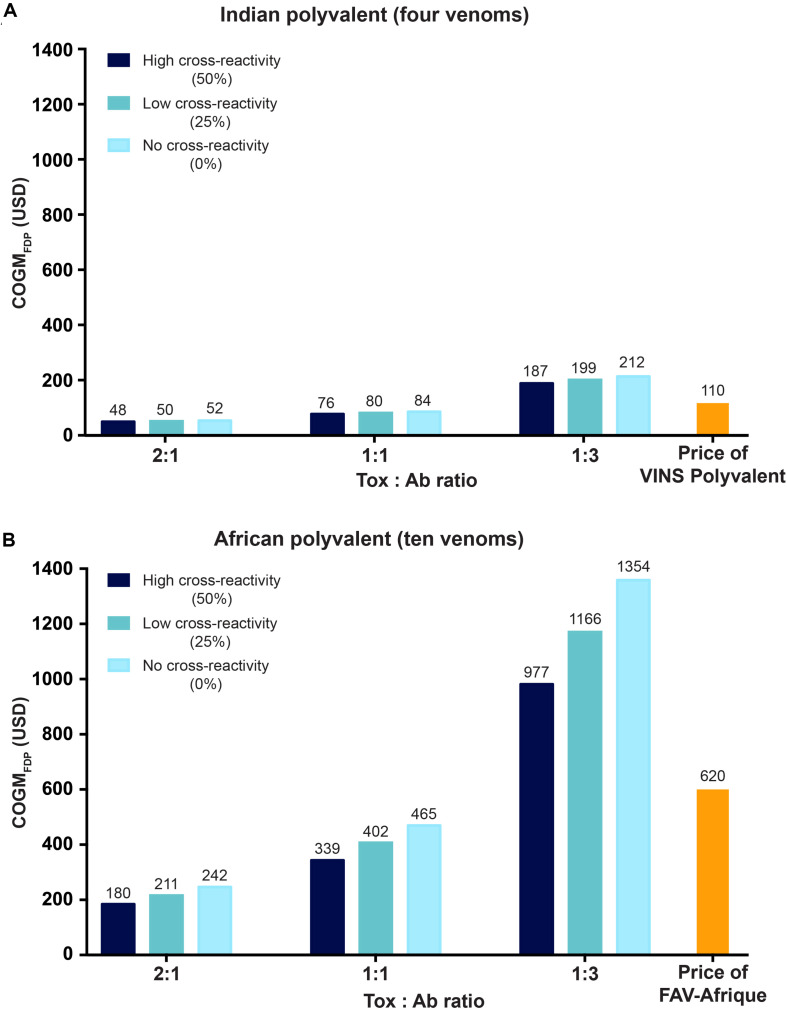
Cost estimates for two polyvalent recombinant antivenoms. **(A)** Putative Cost of Goods Manufactured for the Final Drug Product (COGM_FDP_) for a recombinant antivenom that can neutralize the venoms of the four most medically relevant snakes in India (i.e., *Naja naja*, *Bungarus caeruleus*, *Echis carinatus*, and *Daboia russelii*). **(B)** Cost estimates for a recombinant antivenom that can neutralize 10 different species of snakes in sub-Saharan Africa (i.e., *Bitis arietans, B. gabonica, E. leuconogaster, E. ocellatus, Dendroaspis polylepis, D. jamesoni, D. viridis, N. haje, N. nigricollis*, and *N. melanoleuca*). All of the calculations are conducted for three different toxin-to-antibody ratios (2:1, 1:1, and 1:3). Furthermore, potential cross-reactivity of the monoclonal antibodies present in the recombinant antivenoms against different venom toxins is also included, with estimates including 0% (light blue), 25% (dark turquoise), and 50% (dark blue) cross-reactivity. The costs are calculated for the final drug product, which includes formulation costs. The price per treatment for two animal plasma-derived polyvalent antivenoms for both India (VINS polyvalent) and sub-Saharan Africa (FAV-Afrique – out of production) are also provided for comparison (please note that these are sales prices, which also reflect financial parameters other than COGM alone, such as sales, distribution, indirect costs, and profit margin).

Whilst monovalent antivenoms fulfill an important role in certain regions of the world (such as Australia), polyvalent antivenoms that are effective against a wide range of different venoms are key to solving the global crisis of snakebite envenoming (Gutiérrez et al., 2017). Polyvalent antivenoms eliminate the need for medical practioners to identify the species of venomous snake that bit the patient and, thus, removes the issue of diagnostic uncertainty for the medical practioner (Gutiérrez et al., 2017). The drawback to polyvalent recombinant antivenoms is the complexity of developing them, since it requires that more monoclonal antibodies are included in the formulation of the antivenom, and likely also that the individual antibodies are broadly neutralizing, for the antivenom to be efficacious against many different venoms. To estimate the costs of polyvalent recombinant antivenoms, we explored both a simple antivenom that could neutralize the four most medically relevant snakes in India (i.e., the “Big 4”: *N. naja*, *B. caeruleus*, *D. russelii*, and *E. carinatus*) and a more complex antivenom (10 different venoms from *Dendroaspis* spp., *Bitis* spp., *Naja* spp., and *Echis* spp.) that could be indicated against bites from to the same venomous snakes as a former high-quality antivenom for sub-Saharan Africa (Sanofi Pasteur’s FAV-Afrique). We calculated the costs for very efficacious, efficacious, and less efficacious antibodies, reflected by the toxin-to-antibody ratios (2:1, 1:1, and 1:3, respectively). We also evaluated the impact of antibody cross-reactivity (0%, 25%, and 50%) on the GOGM_FDP_ (Figure 5). Notably, cross-reactivity appears to influence antivenom cost less than antibody efficacy, particularly in the polyvalent recombinant antivenom for the four Indian snakes. However, it appears that the impact of cross-reactivity is significantly higher when assessing more complex and expensive antivenoms, such as the polyvalent recombinant antivenom for sub-Saharan Africa. Additionally, cross-reactivity would simplify the manufacturing process, since less antibodies would need to be produced and quality control would be easier. Consequently, cross-reactivity is likely to have further indirect impact on the COGM_FDP_ than just in the context of the neutralizing capacity of the recombinant antivenom. However, this is not taken into account here due to its rather speculative nature. Nevertheless, the COGM_FDP_ for both polyvalent recombinant antivenoms compare favorably with prices of existing antivenoms. Current Indian polyvalent antivenom costs approximately USD 6.5-11 per vial, with two initial vials being recommended, but 10 vials typically being required (Theakston and Warrell, 1991; Isbister et al., 2015; Alirol et al., 2017). This equates to an antivenom price of USD 13-110 per treatment, which is comparable to both recombinant solutions containing (very) effective antibodies (2:1 and 1:1 toxin-to-antibody), with cost estimates of USD 48-84 per treatment. However, it is of note that this is not taking profit margins into account for the recombinant antivenoms, as well as indirect costs affected by efficacy and safety of treatments are not accounted for here. Similarly, the COGM_FDP_ for a recombinant antivenom appears to compare favorably to the price of the former high-quality polyvalent antivenom for sub-Saharan Africa, FAV-Afrique. Although no longer in production, FAV-Afrique used to be priced between USD 60-140 per vial, and treatments typically required 2–8 vials, resulting in the treatment price ranging from USD 120-1120 (Trop, 2011; Brown, 2012; Harrison et al., 2017). This price is comparable to both recombinant antivenoms containing (very) effective antibodies (2:1 and 1:1 toxin-to-antibody), with cost estimates of USD 180-465 per treatment. Depending on the degree of cross-reactivity, a recombinant antivenom product with less efficacious antibodies (1:3 toxin-to-antibody) would still be less expensive (at 50% cross-reactivity), slightly more expensive (at 25% cross-reactivity), or significantly more expensive (at 0% cross-reactivity). Together, these calculations indicate that polyvalent recombinant antivenoms, even with very broad species coverage, might not only match, but also significantly lower the cost of treatment, whilst likely also providing safer and more efficacious therapy, provided that the antibodies included in the antivenoms are of high therapeutic quality and efficacy.

### Alternative Antitoxins and Their COGM_FDP_

**FIGURE 6:**
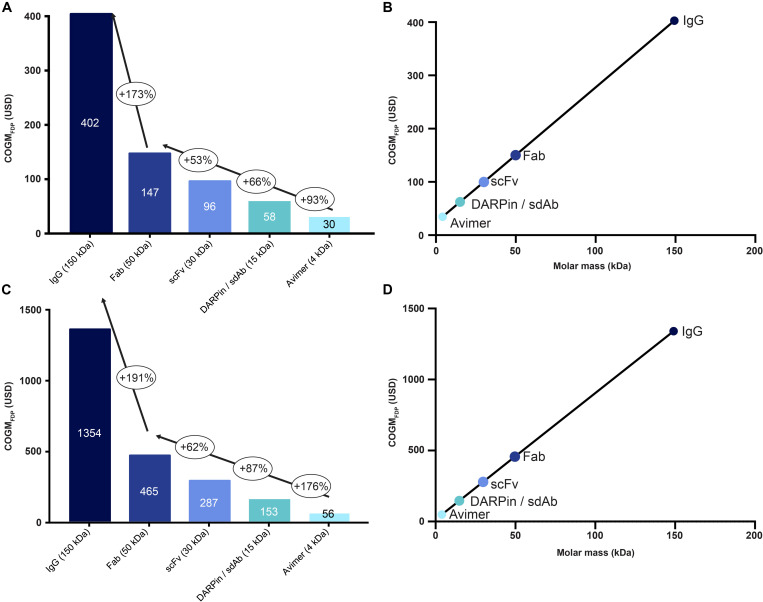
The influence of molar mass of the antitoxin on the cost of recombinant antivenoms for different antibody formats and alternative antitoxin scaffolds. Here, we calculated the cost of a polyvalent recombinant antivenom for sub-Saharan Africa, using an “expected” **(A)** and a “worst case” scenario **(C)** of 1:1 and 1:3 toxin-to-antibody ratio, respectively; the former **(A)** assumes 25% cross-reactivity for the antitoxins, while the latter is calculated without any cross-reactivity. The included formats include IgG, Fab, scFv, DARPin/sdAb, and Avimer. The bubbles indicate the percentage of cost increase from one format to the next and the text in the columns indicate the cost of the final drug product. Correlation between molar masses of the different antitoxin formats and the Cost of Goods Manufactured for the Final Drug Product (COGM_FDP_) of the recombinant antivenom is given for both the “expected” **(B)** and the “worst case” scenario **(D)**, respectively.

IgG antibodies have many advantages, such as a long serum half-life, extensive clinical validation, and established manufacturing strategies. Yet, other smaller formats, including Fabs, scFvs, DARPins, nanobodies, and Avimers, have their own set of advantages (Jenkins et al., 2019; Knudsen et al., 2019). Indeed, these formats have more binding sites per mass unit due to their smaller molar mass, which could have a favorable influence on cost dynamics, as the amount of antitoxin required for neutralizing a given venom may be less (in terms of gram). Consequently, this could lower the final product cost (assuming equimolarity for antivenoms products). Therefore, using the previously mentioned formats we calculated the cost of a polyvalent recombinant antivenom for sub-Saharan Africa, assuming both an “expected” and “worst case scenario” of 1:1 and 1:3 toxin-to-antibody ratios respectively; the former assumes 25% cross-reactivity, while the latter is calculated without any cross-reactivity (Figure 6). We found a major difference in COGM_FDP_ between all scaffolds and a linear relationship between size of the scaffold and the cost of the final drug product. Thus, Avimers were the most inexpensive in both scenarios with an estimated cost of USD 30/56 (expected/worst case) per treatment and IgGs the most expensive with a cost of USD 402/1354 (expected/worst case) per treatment. This demonstrates that even in rare cases where IgGs might not be financially viable, alternative antitoxin scaffolds could be used instead to achieve economic viability. There are, however, other variables to consider when calculating the costs of a recombinant antivenom using alternative antitoxin scaffolds, such as their short half-life (likely requiring administration of larger amounts of the antivenom) and different volumes of distribution (Jenkins et al., 2019). Many alternative antitoxin scaffolds can be produced via microbial expression, rather than mammalian cell cultivation, which may have the potential to be even more cost-competitive at large production volumes. However, given the lack of manufacturing cost data for microbial expression, we decided not to overspeculate in this regard and use the same COGM_API_ for all antitoxin formats (Jenkins et al., 2019). The actual costs for alternative antitoxin scaffolds may, thus, be even more attractive than presented here.

### Limitations of the Study

Whilst the safety and efficacy of any therapeutic should stand at the forefront of all development considerations, it is also key that the product can be manufactured cost-competitively. Indeed, cost of manufacture is of high importance when catering to predominantly low income markets, such as those heavily affected by snakebite envenoming (Harrison et al., 2009). Consequently, we aimed to provide cost estimates for potential recombinant antivenoms to demonstrate that such products are likely to be manufacturable at a cost-competitive level to conventional antivenoms. It is, however, of note that all of our estimates rely on the industry data available to us and the assumptions provided in the methods, such as an expected annual production volume of 500 kg of antibodies. To address this and minimize the impact of incorrect assumptions, we aimed at providing a range of different “scenarios” for most variables included in this study. It should also be noted that the calculations are technical and based on theoretical modeling, which might limit the applicability of the findings to the field. Therefore, the numbers provided here should not be seen as a definitive conclusion to the cost of manufacture for recombinant antivenoms, but rather as a rough guideline toward understanding the cost dynamics at play. Core challenges in improving the accessibility and efficacy of antivenom remain to be resolved in the management of snakebite envenoming.

## Conclusion

New therapeutics often come with exciting treatment prospects for patients. However, it is pivotal to ensure that any new therapy is commercially viable to manufacture and distribute to the market. This is particularly important for antivenoms, which are predominantly required in impoverished regions around the globe. Therefore, in this article, we present the first ever bottom-up cost estimates for recombinant antivenoms. Whilst the numbers should not be taken as definitive conclusions and rather as estimates based on available industry data, the cost dynamics presented here should aid future research and development decisions and strategy. Together, our data indicates that innovative envenoming therapies based on monoclonal antibodies could be manufacturable at a comparable or lower cost to current antivenoms. Indeed, we found that monovalent recombinant antivenoms could be manufactured for USD 20-225 per treatment and more complex polyvalent recombinant antivenoms could be manufactured for USD 48-1354 per treatment. These numbers are slightly higher when compared to previous estimates (USD 33-350 per treatment), yet those calculations were based on a less differentiated top-down approach (Laustsen et al., 2017). Nevertheless, the COGM_FDP_ of recombinant antivenoms falls within a similar spectrum as the prices of currently employed antivenoms (USD 13-1120 per treatment). Finally, manufacturing costs may be even lower for recombinant antivenoms based on alternative antitoxin scaffolds, such as DARPins and nanobodies, which may warrant further research efforts in experimenting with these proteins as putative antitoxins. Given the likelihood of recombinant antivenoms being cost-competitive, alongside their potential therapeutic benefits over conventional antivenoms, further investigation and development of such novel snakebite therapeutics seems warranted.

## Data Availability Statement

The raw data and calculations supporting the conclusions of this article are available upon request to the authors.

## Author Contributions

TJ and AL conceived this study. TJ conducted the analyses and prepared the figures, Both authors drafted and finalized the manuscript.

## Conflict of Interest

The authors declare that the research was conducted in the absence of any commercial or financial relationships that could be construed as a potential conflict of interest.
